# The Vitamin D3 Analog Calcipotriol Attenuates Pancreatic Cancer Malignancy via Downregulating Thrombospondin 1 in Pancreatic Stellate Cells

**DOI:** 10.1155/mi/2632235

**Published:** 2026-04-25

**Authors:** Yang Wu, Chun Zhang, Matthias Ilmer, Maximilian Weniger, Quan Li, Jing Wang, Rainer C. Miksch, Jens Werner, Jan G. D’Haese, Bernhard Renz

**Affiliations:** ^1^ Department of General, Visceral and Transplantation Surgery, Ludwig-Maximilians-University Munich, Munich, Bayern, Germany, uni-muenchen.de; ^2^ Pancreas Center, The First Affiliated Hospital of Nanjing Medical University, Nanjing, Jiangsu, China, njmu.edu.cn; ^3^ The First School of Clinical Medicine, Nanjing University of Chinese Medicine, Nanjing, Jiangsu, China, njucm.edu.cn; ^4^ German Cancer Consortium (DKTK), Partner Site Munich, Munich, Bayern, Germany, dkfz.de; ^5^ Department of General, Visceral and Vascular Surgery, Agatharied Hospital, Hausham, Bayern, Germany

**Keywords:** calcipotriol, CD47, pancreatic cancer, PSCs, THBS1, vitamin D receptor (VDR)

## Abstract

Pancreatic ductal adenocarcinoma (PDAC) exhibits pronounced desmoplasia, primarily attributed to the activation of pancreatic stellate cells (PSCs) from a quiescent state (quiescent PSCs [qPSCs]) to an activated form (activated PSCs [aPSCs]), which facilitates tumor progression and therapeutic resistance. This study investigates the potential of the vitamin D3 (VD) analog calcipotriol (Cal) to modulate this activation process and its impact on PDAC cell malignancy, with a particular focus on the thrombospondin 1/cluster of differentiation 47 (THBS1/CD47) signaling axis. Through analyzing VDR mRNA expression in aPSCs versus PDAC cells, we found that aPSCs are more responsive to VD signaling. Treatment with Cal significantly reduced aPSC activation, as evidenced by decreased α‐SMA expression and THBS1 secretion, thereby diminishing stromal support for PDAC cell proliferation, migration, and invasion. These changes were mediated by the inhibition of the THBS1/CD47 axis, highlighting a novel mechanism by which Cal disrupts the supportive tumor microenvironment. Our findings highlight the therapeutic potential of targeting aPSCs with VD analogs in PDAC, suggesting a new direction for treatments that aim to interrupt the desmoplastic reaction and thereby inhibit PDAC progression.

## 1. Introduction

Pancreatic ductal adenocarcinoma (PDAC) stands as a formidable challenge in oncology, being the 11th most common carcinoma worldwide and the seventh leading cause of cancer‐related mortality with 458,918 new cases and 432,242 deaths in 2018 [1]. Its reputation as one of the deadliest gastrointestinal cancers stems from its aggressive nature and poor response to current therapies [2]. Central to PDAC progression is a pronounced desmoplastic reaction, primarily driven by the activation of pancreatic stellate cells (PSCs) from a quiescent to an activated state [3–5]. Accumulating evidence has proven that this severe fibrogenic stromal reaction plays a vital role in tumor progression and chemotherapy resistance [6]. These cells significantly contribute to the tumor’s fibrotic stroma, thereby facilitating a microenvironment conducive to cancer growth and resistance to chemotherapy [6]. Recent research has highlighted the complex interplay between activated PSCs (aPSCs) and PDAC cells, mediated by paracrine signaling, as a critical factor in cancer progression [6]. This has shifted the therapeutic focus towards modulating the tumor microenvironment, with strategies targeting aPSCs showing promise [5–7]. However, attempts to deplete aPSCs have paradoxically led to more aggressive tumor phenotypes in PDAC transgenic mice, suggesting that nuanced approaches to modulate aPSC activity could offer a more effective therapeutic strategy [6, 8–12].

Detailed information on the proposed strategies can be found in our recently published review [6]. Vitamin D3 (VD) and its analogs have emerged as potential modulators of the tumor microenvironment due to their antifibrotic properties, as evidenced in diseases characterized by significant stromal reactions, such as idiopathic pulmonary and hepatic fibrosis [6, 13–17]. VD is primarily (90%) derived from cutaneous 7‐dehydrocholesterol synthesis induced by ultraviolet radiation (UVR) and partly (10%) from dietary intake [18]. Epidemiological data reveal a lower incidence of PDAC in regions with higher UVR exposure, suggesting a protective role of VD [19, 20]. Additionally, it has been demonstrated that PDAC incidence and mortality rates are negatively correlated with UVR [21–24]. Besides, low expression of VDR or low circulating VD level is associated with poor prognosis of PDAC [25–27]. However, observational studies evaluating the association of PDAC risk and serum levels of 25‐hydroxyvitamin D (25(OH)D) have indicated conflicting results. A pooled analysis of five cohorts suggested that higher circulating levels of 25(OH)D are linked to a lower risk of PDAC [28]. In contrast, a pooled case–control study of eight cohorts in the United States found that a higher 25(OH)D concentration (≥100 nmol/L) is associated with a 2‐fold increase in PDAC susceptibility [29]. Additionally, studies from Egypt and Europe indicated that low serum 25(OH)D is not a risk factor for PDAC [30, 31]. These results underline that VD’s role in PDAC remains uncertain, and further studies exploring the correlation between VD and PDAC are still needed.

Experimental evidence supports the notion that aPSCs, with their robust VDR expression, are prime targets for VD analogs. These compounds have been shown to mitigate stromal fibrosis and enhance the efficacy of chemotherapeutic agents in PDAC models [11, 32]. Notably, VD analogs significantly alter aPSC behavior, including reducing the carcinogenic exosomal production of miR‐10a‐5p, suggesting their potential to disrupt the deleterious crosstalk between aPSCs and PDAC cells [33]. This backdrop of preclinical successes has catalyzed several clinical trials (NCT02754726, NCT03300921, NCT03331562, NCT03415854, NCT03472833, NCT03519308, NCT03520790, and NCT03883919) investigating VD analogs in combination with standard chemotherapies for PDAC treatment.

This study aims to bridge the gap in our understanding of how VD analogs, specifically calcipotriol (Cal), influence the activation state of aPSCs and, by extension, affect the malignancy of PDAC cells through paracrine mechanisms. By dissecting interactions within the tumor microenvironment, we aim to elucidate Cal’s potential to remodel the PDAC stroma, offering insights into novel therapeutic strategies that leverage the modulation of aPSC activity to inhibit PDAC progression.

## 2. Materials and Methods

### 2.1. Isolation and Culture of Primary PSCs and PDAC Organoids

This study was approved by the ethics committee of the First Affiliated Hospital of Nanjing Medical University (Approval No. 2022‐SR‐623). All procedures involving human participants were conducted in accordance with the ethical standards of the institutional research committee and with the Declaration of Helsinki. Written informed consent was obtained from all participants prior to inclusion in the study. After obtaining the approval of the local ethics committee and informed consent, pancreatic tissue was obtained from the biobank of our department and was irreversibly anonymized. aPSCs were isolated from PDAC tumor tissue or chronic pancreatitis (CP) fibrotic tissue via the outgrowth approach [34]. Briefly, the tissue was first trimmed of fatty and connective components. After one PBS wash, the tissue was cut into small pieces (0.5–1 mm^3^) and cultured in six‐well plates (5–10 fragments/well) under standard culture conditions (37°C, 5% CO_2_/air, and humid environment). DMEM/F12 (Gibco, USA), supplemented with 20% fetal bovine serum (FBS) (Sigma–Aldrich, Germany), was used as the culture medium and was renewed every 24 h. After 3–5 days, aPSCs grew from the edge of tissue fragments and could be observed under a light microscope. After PSCs reached a confluence of around 20%, tissue pieces were then removed, and the medium was refreshed twice a week.

Quiescent PSCs (qPSCs) were isolated from adjacent normal pancreatic tissues through density gradient centrifugation [35, 36]. Tissues were first trimmed and then minced and subsequently subjected to enzymatic digestion using a solution of 0.02% pronase, 0.05% collagenase P, and 0.1% DNase (all from Roche, Germany) for 15 min at 37°C. The digest was filtered through a 100 µm cell strainer (BD Bioscience, USA), and cells were washed with Gey’s balanced salt solution (GBSS). The cell suspension was layered over a 28.7% Nycodenz (Axis‐shield PoC, Norway) solution, followed by a careful addition of GBSS with BSA (Biomol, Germany) to form a gradient. After centrifugation at 1400 g for 20 min at 4°C, qPSCs were collected from the interface, cultured in DMEM/F12 with 20% FBS, and stored at 80% confluence.

A standardized culture protocol for PSCs was adopted to ensure optimal cell health and replicative fidelity. The PSCs were cultured in DMEM/F12 supplemented with 10% FBS, 100 units/mL of penicillin, and 100 µg/mL of streptomycin, maintained at 37°C in a humidified atmosphere containing 5% CO_2_.

For PDAC organoids, the tissues were minced and enzymatically dissociated with collagenase IV. The resultant cell suspension was strained through a 40‐μm sieve to remove undigested tissue fragments and then cultured in an organoid‐specific medium. The composition of the organoid culture medium and the culture conditions were adopted as per Sylvia et al., ensuring an optimal environment for organoid growth within a humidified incubator maintained at 37°C and 5% CO_2_ [37]. Organoids were subcultured upon reaching adequate confluency, approximately every 7–10 days.

### 2.2. Cell Lines

Human PDAC cell lines PANC‐1 (male, American Type Culture Collection [ATCC] CRL‐1469, RRID: CVCL_0480), MIA PaCa‐2 (male, ATCC CRL‐1420, RRID: CVCL_0428), and AsPC‐1 (female, ATCC CRL‐1682, RRID: CVCL_0152) were purchased directly from the ATCC (Manassas, VA, USA) in 2015. All lines were maintained in RPMI‐1640 (Gibco, USA) under standard conditions (37°C and 5% CO_2_). Authentication was performed annually by short‐tandem‐repeat (STR) profiling (IDEXX BioResearch, Ludwigsburg, Germany); the latest report (March 2024) showed ≥95% match for each line, and none are listed in the ICLAC Register of Misidentified Cell Lines. Routine mycoplasma screening every 4 months (internal PCR–based SOP) remained consistently negative; the most recent test was completed in June 2024.

### 2.3. qRT‐PCR

Qiagen RNeasy Micro Kits (Qiagen, Germany) and QuantiTect Reverse Transcription Kits (Qiagen, Germany) were used for RNA extraction and cDNA synthesis. qRT‐PCR was performed using SYBR Green and a StepOne real‐time PCR system (Applied Biosystems, Germany). All primers were purchased from Qiagen, and the relative expression of the target gene was calculated using the 2^-ΔCt^ method.

### 2.4. ELISA Assay

The supernatant of PSCs or PDAC was harvested and stored at −80°C. Secretion levels of thrombospondin 1 (THBS1) and TGF‐β in the cell supernatant were examined using commercially available ELISA kits (Human THBS1 Quantikine ELISA Kit, R&D Systems, Germany; Human TGF beta 1 ELISA Kit (ab100647), Germany).

### 2.5. Scratch Assay

For cell migration analysis, an established protocol for wound‐healing assays was used. In brief, cells (5 × 10^5^ cells/well) were seeded into six‐well plates and allowed to reach 100% confluency. Cell monolayers were scratched with a 200 μL tip in every well. After two PBS washes, cells were removed of floating fragments and cultured under different conditions depending on the experiments, as explained below. Pictures were captured at different time points (magnification: 40×). The wound area was analyzed with ImageJ (National Institutes of Health, USA) and used for further statistical analysis.

### 2.6. Transwell Migration and Invasion Assays

The migration and invasion abilities of cells were examined using transwell chambers coated with or without Matrigel (Corning, Germany). Briefly, after culturing in RPMI‐1640 medium (1% FBS) for 24 h, cells (5 × 10^4^) were resuspended in 200 μL FBS‐free medium and added to the upper chamber. A 600 μL volume of medium under different conditions, depending on the experiment, was added to the bottom chamber as a chemoattractant. The cells were allowed to migrate or invade for 24 and 48 h, respectively. After that, the cells on the membrane’s upper surface were removed with cotton swabs. Cells on the lower side were then stained with 0.1% crystal violet (Sigma–Aldrich, Germany) at room temperature for 20 min. Migratory and invasive cells were counted in five high‐power fields (HPFs, magnification: 200×) under a light microscope.

### 2.7. Western Blot

Immunoblots were conducted as previously described [38]. Briefly, total proteins were extracted using a RIPA lysis buffer (Millipore, Germany). Proteins were separated by SDS‐PAGE and transferred to PVDF membranes before incubation with primary antibodies with gentle shaking (2 h, room temperature). The membranes were washed with TBST and incubated with HRP‐conjugated secondary antibodies (room temperature, 2 h). After being washed in TBST, the proteins were visualized using the ChemiDoc MP Imaging System (Bio‐Rad, Germany). Primary antibodies include GAPDH 1:1000 (Cell Signaling Technology, Germany), VDR 1:1000 (Cell Signaling Technology, Germany), α‐SMA 1:1000 (Cell Signaling Technology, Germany), cluster of differentiation 47 (CD47) 1:200 (NOVUS, Germany), vimentin 1:1000 (Cell Signaling Technology, Germany), and E‐cadherin 1:1000 (Cell Signaling Technology, Germany). Secondary antibodies include HRP‐labeled goat antimouse 1:2000 (Santa Cruz Biotechnology, Germany), goat antirabbit 1:2000 (Santa Cruz Biotechnology, Germany), and rabbit antisheep 1:2000 (NOVUS, Germany).

### 2.8. Cell Proliferation Analysis

aPSC proliferation was quantified using an EZ4U kit (Biomedica, Austria). Briefly, cells (1 × 10^4^ cells/well) were seeded in a 96‐well plate with 200 μL of culture medium containing 10% FBS per well. After 24 h, the culture medium was refreshed with a new medium containing different treatments according to the experiments. After 48 h of culture, 20 μL of the dye substrate was added to each well and incubated for 3 h. Finally, the absorbance of cells was determined using a microplate reader set at 450 nm.

For the study of the cell cycle and proliferation in PDAC cell lines PANC‐1 and MIA PaCa‐2, cells were seeded at 350,000 cells per well in six‐well plates and allowed to attach overnight. The medium was then replaced with 2 mL of fresh RPMI supplemented with 10% FBS. For cell cycle analysis, cells were treated with a no‐treatment control (medium only), DMSO control, or 100 nM Cal for 48 h at 37°C. Two hours before completing the incubation, cells were labeled with BrdU using the APC BrdU Flow Kit (BD Biosciences, Cat. No. 557892) for cell cycle analysis. Following treatment, cells were harvested, fixed, and stained according to the kit protocol for DNA content and BrdU incorporation. Analysis was performed using a flow cytometer to evaluate the cell cycle phase distribution and BrdU incorporation, measuring proliferation. Data were analyzed to determine the proportions of cells in G0/G1, S, and G2/M phases, and statistical significance was assessed using one‐way ANOVA; *p*  < 0.05 indicated significance, and all experiments were conducted in triplicate.

### 2.9. Immunocytochemistry (ICC)

ICC was conducted using a commercially available kit (ImmPACT DAB Peroxidase Substrate Kit, Vector Laboratories, Germany). aPSCs were treated with DMSO or 100 nM Cal (Sigma–Aldrich, Germany). A mouse primary antibody (Ab) against α‐SMA 1:250 (Cell Signaling Technology, Germany) was used as the primary Ab, and HRP‐labeled goat antimouse 1:200 (Santa Cruz Biotechnology, Germany) was the secondary Ab. Images were captured under a light microscope (magnification: 100×).

### 2.10. Bioinformatics Analysis

We retrieved the GEO database and utilized three scRNA‐seq datasets (GSE141017, GSE154788, and GSE165399) encompassing normal pancreas, intraductal papillary mucinous neoplasm, and pancreatic carcinoma samples to explore the tumor microenvironment. We further investigated the expression distribution of THBS1 across different cell types and its relationship with PSC states [39–41]. Following the processing workflow for scRNA‐seq data in our previously published article, we processed the scRNA‐seq data using the “Seurat” R package [42]. In brief, firstly, cells with the following criteria were considered passing the quality control: (i) mitochondria UMI rate of <20%, (ii) 200 < nFeature_RNA < 2000, and (iii) nCount_RNA > 1000. Principal component analysis (PCA) was performed on the top 2000 highly variable genes, scaled data, to reduce dimensionality, and the top 10 principal components were used for UMAP reduction. Specific marker genes for each cell type were used to annotate the cells. PSCs with positive expression of α‐SMA/ACTA2 (UMI > 0) were considered aPSCs; otherwise, qPSCs. The Wilcoxon test was used to determine if there is a statistically significant difference in THBS1 expression between aPSCs and qPSCs.

STRING (v11.0, https://string-db.org/) is a tool for analyzing gene relationships and establishing protein–protein interaction networks [43]. For the current study, PubMed was searched for studies on differentially expressed genes during PSC activation. Then, the protein–protein interaction networks among these genes were generated using the STRING database. GEPIA (http://gepia.cancer-pku.cn/index.html) is a tool for analyzing RNA sequencing expression data. It provides functions for analyzing gene expression, correlation, and patient survival based on data from the tumor and paired normal samples in the Cancer Genome Atlas (TCGA) and the Genotype‐Tissue Expression (GTEx) databases [44]. THBS1 and CD47 mRNA expression in PDAC (*n* = 179) and paired normal tissue (*n* = 171) was investigated using GEPIA. Survival analyses of THBS1 and CD47 in PDAC patients were also conducted using this online tool.

### 2.11. Statistical Analysis

Statistical analysis was done using SPSS 21.0. The Student’s *t*‐test was used to analyze normally distributed data; for non‐normally distributed data, the Mann–Whitney *U* test was used. For single‐cell sequencing data analysis and related visualization, RStudio (version 4.2.0) and relevant R packages were utilized. Chi‐squared tests were used to evaluate the correlation between VDR and α‐SMA expression. Data were described as mean ± SD. Each experiment was performed at least three times in triplicate, and *p*  < 0.05 was considered statistically significant.

## 3. Results

### 3.1. Selective Inhibition of aPSC Activation by Cal in PDAC

**Figure 1:**
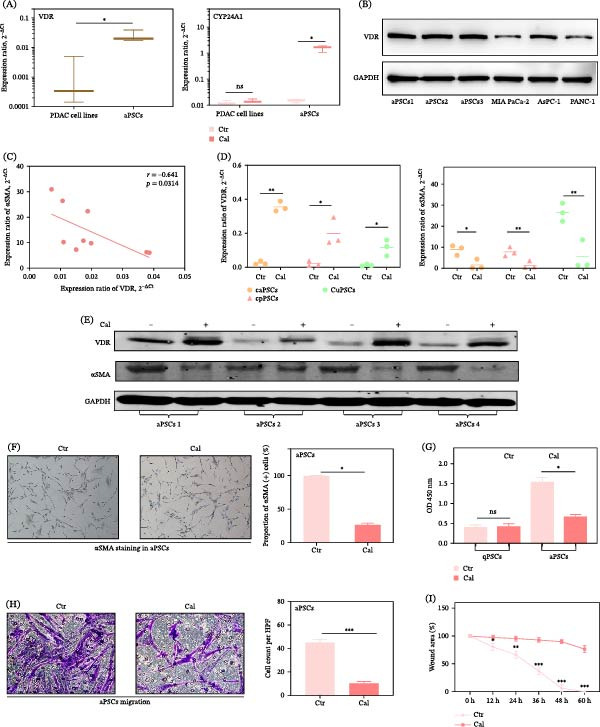
Effects of Cal on aPSC activation. (A) (Left) VDR mRNA expression in PDAC cell lines (AsPC‐1, MIA PaCa‐2, and PANC‐1) and aPSCs was determined by qRT‐PCR (*n* = 3). (Right) CYP24A1 mRNA expression in PDAC or aPSCs treated with DMSO or Cal (100 nM and 48 h) was examined by qRT‐PCR (*n* = 3). (B) VDR protein expression in PDAC cell lines (AsPC‐1, MIA PaCa‐2, and PANC‐1) and aPSCs was determined by western blot (*n* = 3). (C) Correlation analysis between α‐SMA and VDR mRNA expression in aPSCs, with GAPDH normalization (*n* = 9). (D) VDR and α‐SMA gene expression in aPSCs treated with DMSO or Cal (100 nM and 48 h) was evaluated by qRT‐PCR (*n* = 3). (E) VDR and α‐SMA protein expression in aPSCs treated with DMSO or Cal (100 nM and 48 h) (*n* = 4). (F) Immunocytochemistry showing α‐SMA expression in aPSCs treated with DMSO or Cal (100 nM and 48 hr) (*n* = 3). (G) EZ4U assay indicating the impacts of Cal on the proliferation of aPSCs (*n* = 3). (H) Transwell migration assay and (I) wound healing showing the effects of Cal on aPSCs’ migration ability (*n* = 3). caPSCs, PSCs derived from pancreatic cancer; cpPSCs, PSCs derived from chronic pancreatitis; cuPSCs, culture‐activated PSCs derived from normal tissue; aPSCs, activated PSCs; HPF, high‐power field; Ctr, control group treated with DMSO. All experiments were conducted in triplicate. ns, not significant.  ^∗^ *p*  < 0.05,  ^∗∗^ *p*  < 0.01, and  ^∗∗∗^ *p*  < 0.001.

VDR signaling plays a crucial role in aPSC biology, with implications for PDAC progression. Our investigation began with an analysis of VDR expression in aPSCs compared to PDAC cell lines. We observed that aPSCs express higher levels of VDR mRNA, indicating an enhanced sensitivity to vitamin D signaling modulation by Cal. The VDR activity, as measured by the induction of CYP24A1 mRNA [45], was markedly enhanced following Cal treatment in aPSCs, suggesting an activation of vitamin D metabolism. However, this response was not observed in PDAC cells, highlighting the specificity of Cal’s action on aPSCs (Figure 1A,B and Figure S1A,B).

A moderate inverse correlation between VDR and α‐SMA expression was observed, consistent with the proposed antifibrotic role of VDR (Figure 1C). Further investigation into Cal’s effects revealed its capacity to significantly reduce α‐SMA levels across various aPSC subtypes, including those associated with cancer (caPSCs), CP (cpPSCs), and culture‐activated PSCs from normal tissues (cuPSCs). This consistent reduction in α‐SMA, irrespective of cell origin, highlights Cal’s broad‐spectrum potential to suppress fibrogenic activity across different aPSC populations, underscoring its therapeutic value in addressing the stromal aspects of the tumor environment (Figure 1D).

Further evaluation of Cal’s impact on aPSCs revealed significant downregulation of α‐SMA at both mRNA and protein levels, indicating the deactivation of fibrotic features in aPSCs (Figure 1D‐F). Functional assays further validated these findings, showing a significant reduction in aPSCs’ motility and proliferation following Cal treatment, as evidenced by EZ4U, scratch, and transwell migration assays (Figure 1G‐I). Additional functional analyses, including migration and invasion assays, further highlighted Cal’s aPSCs‐specific inhibitory effects, which were not observed in PDAC cells, thus reaffirming Cal’s targeted mechanism of action (Figure S1C,D). Flow cytometry analysis corroborated these findings, showing no alteration in the proliferation capabilities of PANC‐1 and MIA PaCa‐2 cells post‐Cal treatment, further distinguishing the selective reactivity of aPSCs to Cal (Figure S1E). These results suggest that Cal selectively targets aPSCs within the complex PDAC environment, focusing on influencing the surrounding stromal cells rather than directly affecting cancer cells.

### 3.2. THBS1 as a Prognostic Marker and Its Elevated Levels in aPSCs

To identify the highest‐risk secreted proteins associated with aPSCs, we established four stringent criteria to select our final target genes. As depicted in the Venn diagram in Figure 2A, we labeled our screening categories: “secretome,” “biomarkers of poor prognosis,” “upregulated in aPSCs,” and transcripts with a fold change (FC) > 1.5 in aPSCs after Cal treatment [11, 46]. Consequently, THBS1 emerged as the prime candidate for subsequent research. Within the TCGA‐PAAD cohort, THBS1 expression was identified as a significant biomarker correlating with progression‐free and overall survival, indicating an unfavorable prognosis (Figure 2B). Utilizing the aforementioned scRNA‐seq datasets [39–41], we then mapped THBS1 expression across various cell types in the pancreatic TME. We noted the specific expression of THBS1 in PSCs, as indicated by the blue‐dotted cluster in the UMAP plots (Figure 2C), and subsequent blue boxplots showed that it was elevated in PSCs compared to other cell populations (Figure 2D). Expectedly, similar results were observed in datasets GSE154778 and GSE165399 (Figure 2E‐H). Moreover, we categorized PSCs into aPSCs and qPSCs based on α‐SMA expression across the three scRNA‐seq datasets and observed significantly higher THBS1 expression in aPSCs than in qPSCs. Collectively, these bioinformatics analyses suggest that THBS1 is a risk‐associated secreted protein predominantly expressed in aPSCs in PDAC (Figure 2I and Figure S2A,B).

Figure 2aPSC‐derived THBS1 as a prognostic marker in PDAC. (A) A Venn diagram illustrates THBS1 as the unique gene that intersects with the activation of aPSCs, secretome enrichment, biomarkers of poor prognosis, and responsiveness to Cal treatment. (B) Survival analysis showing high THBS expression is linked to lower progression‐free and overall survival in PDAC patients. (C–H) The differential expression of THBS1 across various cell types in the tumor microenvironment using single‐cell RNA sequencing data from the datasets (GSE141017, GSE154778, and GSE165399). (I) Single‐cell RNA‐seq analysis reveals elevated THBS1 expression in aPSCs compared to qPSCs. (J) ELISA data demonstrate the levels of THBS1 protein in the supernatant of PDAC cells, cuPSCs, cPSCs, and caPSCs, indicating elevated THBS1 in the PDAC context. (K) THBS1 protein quantification in supernatants of control and Cal‐treated aPSCs from different origins, highlighting the decrease post‐treatment. All experiments were conducted in triplicate.  ^∗^ *p*  < 0.05,  ^∗∗^ *p*  < 0.01,  ^∗∗∗^ *p*  < 0.001, and  ^∗∗∗∗^ *p*  < 0.0001.

**Figure:**
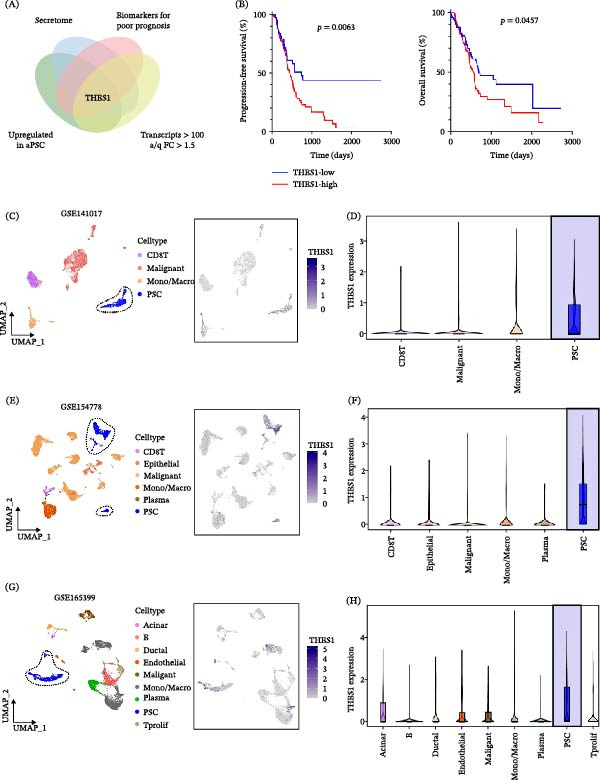

**Figure:**
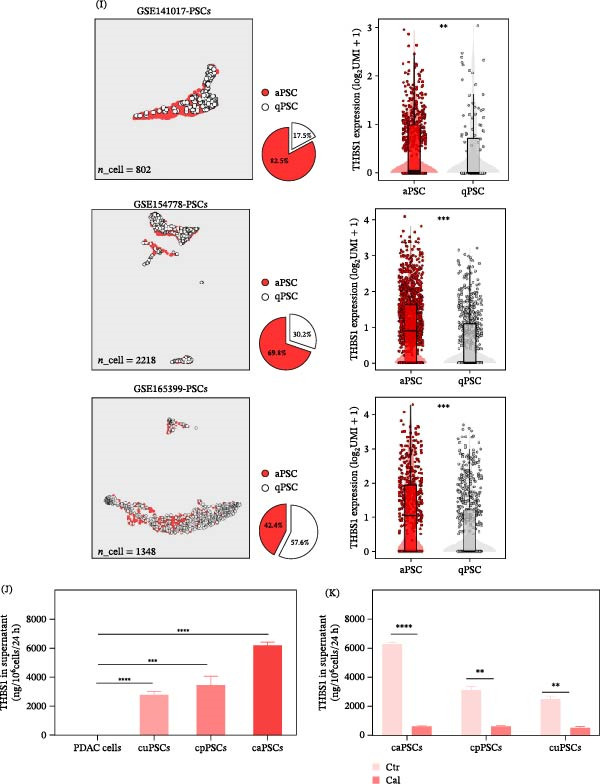

ELISA validation, as shown in Figure 2J, corroborated these findings, demonstrating elevated THBS1 levels in aPSC supernatants. Moreover, while TGF‐β protein levels in aPSC supernatants remained unchanged after Cal treatment, THBS1 levels significantly decreased, pinpointing THBS1 as a more specific therapeutic target (Figure 2K and Figure S2C). This finding aligns with the recognized role of aPSCs as key contributors to the protumorigenic milieu through the secretion of bioactive molecules. This sequential unraveling of THBS1 dynamics offers novel insights into targeting stromal elements of PDAC to improve patient outcomes.

### 3.3. Dose‐Dependent Augmentation of PDAC Aggressiveness by THBS1

THBS1 has been recognized for its role in intensifying the aggressiveness of various cancers, including those of the thyroid, breast, and prostate [47–51]. However, its impact on PDAC malignancy remained unclear, prompting an investigation into THBS1’s effects on PDAC cells, but its effect on the malignancy of PDAC is still not apparent.

**Figure 3:**
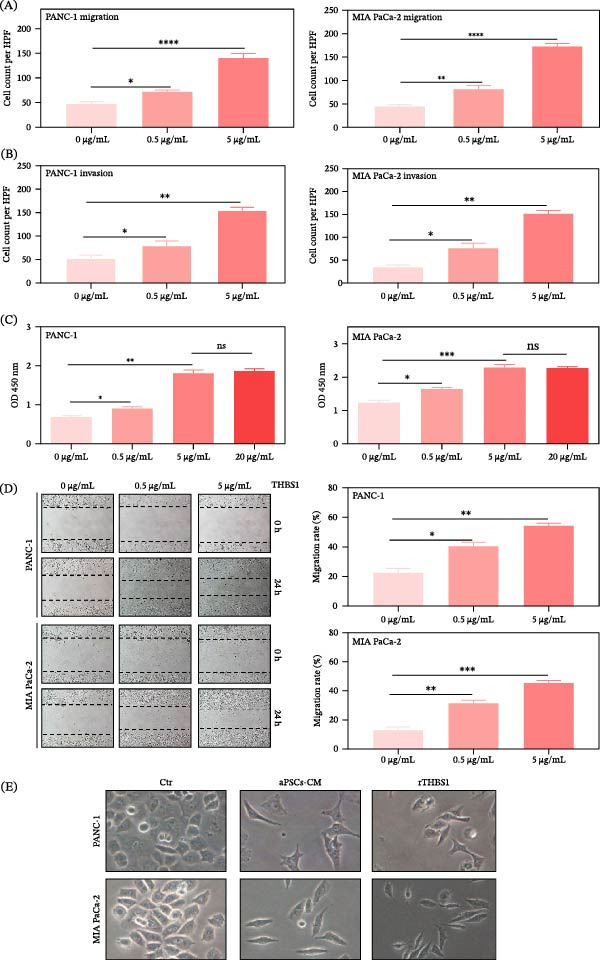
Dose‐dependent effect of rTHBS1 on PDAC malignancy. (A) Transwell migration assays showing the response of PDAC cell lines PANC‐1 and MIA PaCa‐2 to varying concentrations of rTHBS1 (0, 0.5, and 5 μg/mL). (B) Transwell invasion assays were used to quantify the invasive potential of the same PDAC cell lines under the same rTHBS1 treatments. (C) Proliferation of PDAC cells was assessed by EZ4U assay after treatment with rTHBS1 at 0, 0.5, 5, and 20 μg/mL. (D) Wound healing assays complement the migration analysis, with images and quantification of the migration area closure. (E) Representative micrographs depicting morphological alterations in PANC‐1 and MIA PaCa‐2 cells when cultured in standard medium, aPSCs‐CM, and standard medium supplemented with 5 μg/mL of rTHBS1. All experiments were conducted in triplicate.  ^∗^ *p*  < 0.05,  ^∗∗^ *p*  < 0.01,  ^∗∗∗^ *p*  < 0.001, and  ^∗∗∗∗^ *p*  < 0.0001.

In a series of dose–response experiments, recombinant THBS1 (rTHBS1) was shown to progressively enhance the malignancy characteristics of PDAC cell lines. Specifically, transwell migration assays (Figure 3A) showed a dose‐dependent increase in migratory ability for PANC‐1 and MIA PaCa‐2 cells, correlating with rising rTHBS1 levels. This was also evident in invasion assays (Figure 3B), where invasive potential increased with increasing rTHBS1. A marked enhancement in the proliferation of the PANC‐1 and MIA PaCa‐2 cell lines was also observed, with a direct relationship to rTHBS1 dosage. Notably, this proliferative uptick reached its zenith at a 5 μg/mL concentration, where it plateaued, indicating a dose–response saturation point (Figure 3C). This observation prompted the selection of 5 μg/mL rTHBS1 for subsequent analyses to explore its effect on PDAC cell behavior comprehensively. Further analysis using wound‐healing assays (Figure 3D) supported these findings, showing accelerated migratory capacity in response to rTHBS1. Morphological observations revealed that PDAC cells underwent epithelial‐mesenchymal transition (EMT)–like changes upon exposure to rTHBS1, adopting a spindle‐shaped appearance indicative of a mesenchymal phenotype (Figure 3E).

### 3.4. Cal Reduces aPSC‐Mediated PDAC Proliferation, Invasion, Migration, and EMT by Downregulating THBS1/CD47

Furthering our understanding of THBS1’s role in PDAC aggressiveness, we investigated Cal’s capability to influence PDAC cell dynamics by targeting the tumor microenvironment shaped by aPSCs. By incorporating a THBS1‐neutralizing Ab, we aimed to dissect the role of this stromal protein in cancer progression, anticipating that its inhibition would disrupt promalignant signaling pathways and reveal potential therapeutic targets within the complex interplay between PDAC cells and their surrounding stroma.

**Figure 4:**
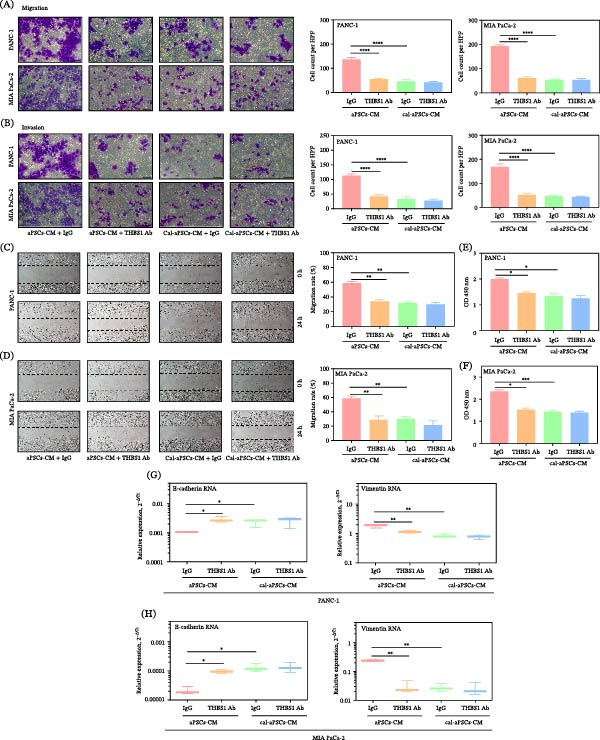
Inhibition of aPSCs‐CM–driven malignancy in PDAC by THBS1 neutralizing antibody. THBS1 neutralizing Ab diminished aPSCs‐CM–induced migration (A, C, D), invasion (B), proliferation (E–F), and EMT (G–H) of PDAC but had no effects on Cal‐aPSCs‐CM–induced malignancy of PDAC. aPSCs‐CM, CM from aPSCs pretreated with DMSO; Cal‐aPSCs‐CM, CM harvested from aPSCs pretreated with 100 nM Cal for 48 h. CM was then pretreated with 1 μg/mL of THBS1 Ab or control IgG and added to the PDAC.  ^∗^ *p*  < 0.05,  ^∗∗^ *p*  < 0.01,  ^∗∗∗^ *p*  < 0.001, and  ^∗∗∗∗^ *p*  < 0.0001.

To determine whether Cal modulates aPSC‐induced malignancy in cancer cells by regulating THBS1 expression, we depleted THBS1 in CM from aPSCs or Cal‐aPSCs (aPSCs pretreated with Cal) using a neutralizing Ab and added the CM to PDAC. Results demonstrated that neutralizing THBS1 significantly tempered the malignancy‐enhancing impact of aPSCs‐CM on PDAC cells, evidenced by reduced migration and invasion in transwell assays (Figure 4A,B), and diminished migration rates in wound healing assays (Figure 4C,D).

Moreover, the proliferation of PDAC cells, as assessed by EZ4U assays, was markedly diminished when cultured with THBS1‐depleted aPSCs‐CM, underscoring the importance of THBS1 in aPSCs‐CM–mediated promotion of PDAC cell growth (Figure 4E,F). Analysis of EMT markers revealed that the neutralizing Ab effectively downregulated mesenchymal markers and upregulated epithelial markers, suggesting reversal of the EMT process (Figure 4G,H).

Based on THBS1’s role in enhancing PDAC malignancy, we explored the integral role of CD47, THBS1’s receptor, in mediating these effects [52]. This exploration was crucial for deciphering the intricate mechanisms by which aPSCs‐CM affects PDAC cells, with a focus on the THBS1–CD47 interaction as a key driver of PDAC progression and a potential therapeutic target.

**Figure 5:**
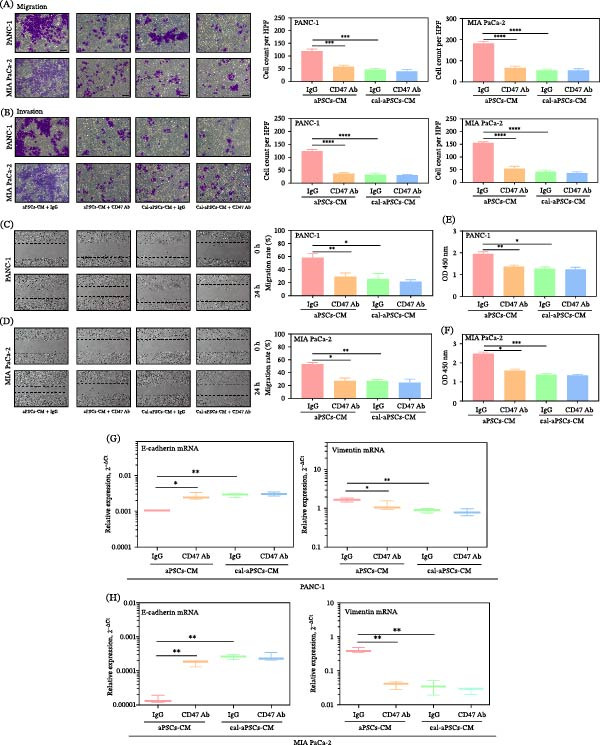
Attenuation of aPSCs‐CM–induced PDAC aggressiveness by CD47 blockade. CD47 blocking Ab diminished aPSCs‐CM–induced migration (A, C, D), invasion (B), proliferation (E–F), and EMT (G–H) of PDAC but had no effects on Cal‐aPSCs‐CM–induced aggressiveness of PDAC. PDAC were pretreated with 2 μg/mL CD47 blocking Ab or control IgG. All experiments were conducted in triplicate.  ^∗^ *p*  < 0.05,  ^∗∗^ *p*  < 0.01,  ^∗∗∗^ *p*  < 0.001, and  ^∗∗∗∗^ *p*  < 0.0001.

The therapeutic potential of CD47 blockade in modulating the malignancy induced by aPSCs‐CM in PDAC cells was investigated. Transcriptional analysis using the GEPIA online tool showed that CD47 is overexpressed in pancreatic cancer tissues compared to adjacent normal tissues, and this overexpression is negatively correlated with disease‐free survival (Figure S2D). Additionally, western blot validation (Figure S2E) confirmed that CD47 protein expression is elevated in tumor tissues compared to adjacent normal tissues. Investigations into CD47’s therapeutic potential have revealed its enhanced expression in PDAC tumor tissues, indicating its role as a “don’t eat me” signal that facilitates cancer cell evasion from immune destruction. Blocking CD47 significantly mitigated the migratory and invasive responses of PDAC cells to aPSCs‐CM in migration and invasion assays (Figure 5A‐D). Proliferation assays confirmed the suppression of PDAC cell growth (Figure 5E,F). Quantitative PCR of EMT markers post‐CD47 blockade showed decreased mesenchymal markers and increased epithelial markers, indicating a potential EMT reversal (Figure 5G,H). This indicated that the protumorigenic activity of aPSCs‐CM is, at least in part, mediated through the THBS1–CD47 interaction.

Additionally, it has been reported that aPSCs can increase PDAC invasiveness by reducing the L1CAM expression in tumor cells [53]. Therefore, we measured the L1CAM mRNA expression in pancreatic cancer cell lines PANC‐1 and MIA PaCa‐2 treated with Cal‐aPSCs‐CM. However, we found no statistically significant difference in the L1CAM mRNA expression in pancreatic cancer cells (Figure S2F). This may be because L1CAM expression in pancreatic cancer cells is influenced by other components in the aPSCs‐CM, and Cal pretreatment of aPSCs‐CM may not be effective in this regard.

### 3.5. Modulation of PDAC Organoid Characteristics by aPSCs‐CM and Targeted Therapies

Based on discoveries regarding the THBS1/CD47 interaction and Cal’s regulatory effects, we extended our investigation to organoid models. This approach aimed to demonstrate concretely how molecular interactions influence tumor behavior, thereby advancing our understanding of viable PDAC treatment strategies. Transitioning from molecular insights to tangible applications underscores our holistic approach to identifying efficacious PDAC interventions. We examined the effects of various treatments on the morphology and expression of EMT markers in PDAC organoids over 5 days. The treatments included control (aPSCs‐CM), Cal‐aPSCs‐CM, THBS1 Ab–depleted aPSCs‐CM, and CD47 Ab–treated organoids followed by aPSCs‐CM exposure.

**Figure 6:**
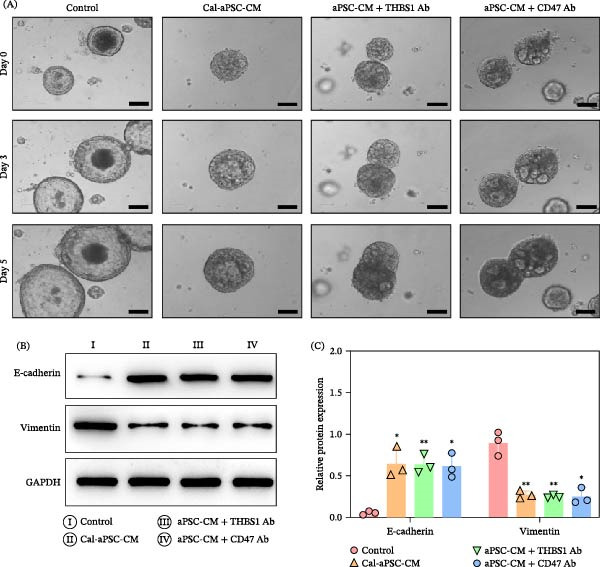
Differential impact on PDAC organoid morphology and EMT marker expression by aPSCs‐CM and antibody interventions. (A) Representative bright‐field images displaying PDAC organoids over 5 days in control (aPSCs‐CM), treated with Cal‐aPSCs‐CM, with THBS1 antibody‐depleted aPSCs‐CM, and with organoids where CD47 has been blocked, followed by treatment with aPSCs‐CM. (B) Western blot analysis of E‐cadherin and vimentin in organoids subjected to these varied treatments. (C) Protein expression quantification normalized to GAPDH, demonstrating the effect of THBS1 depletion and CD47 inhibition on EMT markers in PDAC organoids. All experiments were conducted in triplicate.  ^∗^ *p*  < 0.05 and  ^∗∗^ *p*  < 0.01. Scale bar: 100 μm.

Patient‐derived organoids (PDOs) are recognized as a sophisticated model for replicating the three‐dimensional architecture and functionality of PDAC within a setting that closely mimics in vivo conditions [37, 53]. Our results showed that, compared to the control group, which exhibited significant growth, PDOs treated with Cal‐aPSCs‐CM, THBS1 Ab–depleted aPSCs‐CM, or organoids pretreated with a CD47‐blocking Ab before exposure to aPSCs‐CM displayed minimal proliferation within the same timeframe (Figure 6A).

Western blot analysis (Figure 6B) and quantification (Figure 6C) of EMT markers revealed that organoids treated with Cal‐aPSCs‐CM or THBS1 Ab–depleted aPSCs‐CM showed an increase in the epithelial marker E‐cadherin and a decrease in the mesenchymal marker vimentin, indicating a reversal of EMT. Similarly, organoids pretreated with a CD47‐blocking Ab before exposure to aPSCs‐CM also showed changes in EMT marker expression, consistent with a shift toward a more epithelial phenotype (Figure 6B,C).

## 4. Discussion

The complex interplay between aPSCs and PDAC cells is a cornerstone of PDAC progression [54]. This study examines the intricate dynamics of this interaction, focusing on the transformative potential of reprogramming aPSCs using the VD analog Cal as a novel approach to modulate PDAC progression [55]. Our research shows three significant findings: the role of THBS1 secretion by aPSCs in enhancing PDAC aggressiveness, the inhibition of aPSCs activation by Cal, and the attenuation of aPSC‐driven PDAC malignancy via the suppression of the THBS1/CD47 signaling pathway.

Our findings align with previous research, demonstrating distinct VDR expression and function in aPSCs compared to PDAC cells, underscoring their differential response to VD signaling modifications [11]. Treatment with Cal decreased α‐SMA expression and reduced migration and proliferation of aPSCs, consistent with the quiescence induced by VD analogs documented in related studies [11, 32, 56, 57]. This suggests that through VDR modulation, VD analogs can reprogram aPSCs, potentially slowing the advancement of PDAC. Importantly, our mechanistic findings offer a resolution to broader clinical controversies.

Indeed, while aPSCs have been shown to modulate the biology of cancer cells through various signaling molecules [58–66], little is known about the mechanism by which VD influences the interaction between aPSCs and PDAC. Our investigation into the THBS1/CD47 axis offers novel insights into the protumorigenic effects mediated by aPSCs within the PDAC microenvironment. THBS1, a 450‐kDa ECM protein, emerges as a critical modulator of tumor behavior, primarily through its interaction with CD47, facilitating aggressive tumor phenotypes across various cancers [67–69]. Interestingly, recent studies have indicated that THBS1 is highly expressed in the stromal area of PDAC [70, 71]. It has also been shown that fibroblasts secrete THBS1 at significantly higher levels than tumor cells in various cancers [72–76]. Our study corroborates and extends previous findings by demonstrating elevated THBS1 secretion in aPSCs compared with PDAC cells, with tumor‐derived aPSCs as the predominant source. This significant release of THBS1 by aPSCs underscores the pivotal role of the tumor microenvironment, particularly stromal cells, in driving PDAC progression. The ligand‐receptor duo of THBS1 and CD47 was reported to promote metastasis and aggressiveness in several tumor types [49–51, 67, 69, 77–80]. Okada et al. [81] revealed that high stromal THBS1 expression correlates with poor prognosis and invasiveness in intraductal papillary mucinous neoplasms of the pancreas. However, little is known about THBS1–CD47 signaling in PDAC. The association between high stromal THBS1 expression and poor prognosis in PDAC patients, as highlighted in our results, aligns with observations in other tumor types, reinforcing the critical nature of the THBS1/CD47 axis in cancer aggressiveness. Moreover, our findings that THBS1 induces proliferation, migration, invasion, and EMT in PDAC cells further affirm the axis’s tumor‐promoting role. The inhibitory effects observed upon blockade of the THBS1/CD47 interaction provide compelling evidence for its contribution to aPSC‐mediated malignancy in PDAC, presenting a viable target for therapeutic intervention.

While epidemiological studies on vitamin D status and PDAC risk have shown inconsistencies, the association between low VDR expression/poor vitamin D status and worse patient prognosis is more consistent. Our mechanistic discovery that the VDR agonist Cal effectively reprograms the protumorigenic aPSCs provides a plausible biological basis for these clinical observations, shifting the focus from systemic epidemiology to a localized, stromal‐targeted therapeutic strategy.

The potential for clinical translation of our findings is significant, offering a promising avenue for developing novel PDAC therapies. Recent studies supporting the efficacy of THBS1 or CD47 blockade in reducing tumor growth and metastasis in murine models of PDAC complement our in vitro observations, suggesting a crucial role for the THBS1/CD47 axis in stroma–tumor interactions [70, 82–84]. The combination of CD47 inhibition with chemotherapy, as reported to enhance treatment outcomes in PDAC models, underscores the synergistic potential of targeting this axis in conjunction with conventional therapies [85]. Our findings supplement the existing literature by clarifying the role of the THBS1/CD47 axis in promoting tumor aggressiveness mediated by aPSCs within the PDAC microenvironment. However, further discussion is warranted to understand the significance of these mechanisms in PDAC biology and their position within the broader molecular landscape of PDAC. Comparing these findings with other known signaling pathways in PDAC could offer a more comprehensive biological insight and reveal potential therapeutic targets.

Despite these advancements, the path to clinical application requires addressing several challenges, including optimizing drug delivery systems, ensuring treatment specificity, and overcoming resistance mechanisms. The variability in patient and tumor responses to VD analogs stresses the need for targeted research to discover predictive biomarkers for more personalized treatment strategies. This precision medicine approach is crucial for refining clinical trials and advancing the management of PDAC.

Moreover, we acknowledge the limitations of using specific PDAC cell lines and activation and treatment conditions for aPSCs, which may not fully represent the heterogeneity of PDAC. Future research should aim to validate and expand our findings across a broader range of PDAC models and clinical samples and to explore combination therapies that address the complexity of PDAC progression and treatment resistance.

In summary, our study emphasizes the innovative potential of targeting aPSCs to influence PDAC progression, highlighting the therapeutic promise of VD analogs in reprogramming aPSCs to mitigate tumor aggressiveness. A multidisciplinary effort will be necessary to translate these findings into clinical strategies, overcoming current challenges and maximizing the therapeutic potential of aPSC‐targeted therapies in PDAC treatment. Future investigations should focus on uncovering additional molecular targets; evaluating the effectiveness of combination therapies involving VD analogs, THBS1/CD47 blockade, and conventional treatments; and understanding the impact of genetic and environmental factors on therapeutic responses. This comprehensive approach is vital for developing tailored treatment strategies in the fight against PDAC.

## 5. Conclusions

Our study reveals a critical role for aPSCs in promoting PDAC progression through THBS1 secretion, identifying the THBS1/CD47 axis as a key player in this process. The use of the vitamin D analog, Cal, effectively inhibits aPSC activation and disrupts the malignancy‐enhancing THBS1/CD47 signaling, presenting a novel therapeutic strategy against PDAC by targeting its microenvironment rather than the cancer cells directly. These findings open up promising avenues for the development of targeted therapies that modulate the tumor microenvironment, offering hope for improved treatments for patients with PDAC.

## Author Contributions

**Yang Wu:** conceptualization, methodology, investigation, data curation, formal analysis, writing – original draft, project administration. **Chun Zhang:** investigation, methodology, validation, data collection, writing – review and editing. **Matthias Ilmer:** methodology, supervision, resources, writing – review and editing. **Maximilian Weniger:** formal analysis, validation, writing – review and editing. **Quan Li:** investigation, data collection, writing – review and editing. **Jing Wang:** investigation, experimental support, data curation, writing – review and editing. **Rainer C. Miksch:** technical support, resources, writing – review and editing. **Jens Werner:** supervision, resources, writing – review and editing. **Jan G. D’Haese:** conceptualization, supervision, project oversight, writing – review and editing. **Bernhard Renz:** supervision, project oversight, scientific input, writing – review and editing.

## Funding

This study was funded by the Chinese Scholarship Council (Grants 201708320343, 201708320342, JSSCBS20221832, and JSSCBS20220472) and the National Natural Science Foundation of China (Grant 82304902). Open Access funding enabled and organized by Projekt DEAL.

## Disclosure

All authors have read the final version and agreed to the submission.

## Conflicts of Interest

The authors declare no conflicts of interest.

## Supporting Information

Additional supporting information can be found online in the Supporting Information section.

## Supporting information


**Supporting Information** Figure S1: Evaluation of VDR expression and functional assays in PDAC cell lines after Cal treatment. (A) Quantification of VDR expression in PANC‐1 cells treated with control (RPMI1640), DMSO, or 100 nM Cal. (B) Quantification of VDR expression in MIA PaCa‐2 cells under similar treatment conditions (*n* = 3). (C) Migration assay results for PANC‐1 and MIA PaCa‐2 cells, showing cell counts per highipower field (HPF) with or without Cal treatment (*n* = 3). (D) Invasion assay results for the same cell lines, indicating no significant difference in cell count per HPF between treatments (*n* = 3). (E) Flow cytometry analysis of the cell cycle distribution and proliferation in PDAC cell lines treated with DMSO or Cal. All experiments were conducted in triplicate. ns: not significant,  ^∗^ *p*  < 0.05. Figure S2: THBS1 and CD47 expression and survival analysis in PDAC. (A) A protein‐interaction network illustrates the connections between THBS1, VDR, and other proteins. (B) Using GEPIA, transcriptomic analysis of PDAC tumor samples reveals the expression of THBS1 across a cohort, with a box plot indicating a correlation between high THBS1 levels and decreased disease‐free survival. (C) ELISA quantification of TGF‐β protein levels in the supernatant of aPSCs treated with medium (Ctr), DMSO, or 100 nM Cal, showing no significant differences. (D) Gene expression profile for CD47 across the same PDAC cohort, as analyzed using GEPIA, with a box plot illustrating the association between high CD47 levels and reduced disease‐free survival. (E) Western blot analysis of CD47 protein levels in PDAC tumor tissue (T) and adjacent normal tissue (N) from patients, with densitometry confirming higher CD47 expression in tumor tissue (*n* = 3). (F) qRT‐PCR analysis of LCAM expression in PDAC cells treated with aPSCs‐CM (Ctr), DMSO‐aPSCs‐CM, or Cal‐aPSCs‐CM, indicating no significant change in expression due to Cal treatment (*n* = 3). All experiments were conducted in triplicate. ns: not significant,  ^∗^ *p*  < 0.05.

## Data Availability

The data that support the findings of this study are available from the corresponding author upon reasonable request.
