# Supplementary material for: The Vitamin D3 Analog Calcipotriol Attenuates Pancreatic Cancer Malignancy via Downregulating Thrombospondin 1 in Pancreatic Stellate Cells
Source: Mediators Inflamm. 2026 Apr 25;2026:2632235. doi: 10.1155/mi/2632235 (PMC13109869; doi:10.1155/mi/2632235)
Supplement: Supplementary file 1 — Supporting Information Figure S1: Evaluation of VDR expression and functional assays in PDAC cell lines after Cal treatment. (A) Quantification of VDR expression in PANC‐1 cells treated with control (RPMI1640), DMSO, or 100 nM Cal. (B) Quantification of VDR expression in MIA PaCa‐2 cells under similar treatment conditions (n = 3). (C) Migration assay results for PANC‐1 and MIA PaCa‐2 cells, showing cell counts per highipower field (HPF) with or without Cal treatment (n = 3). (D) Invasion assay results for the same cell lines, indicating no significant difference in cell count per HPF between treatments (n = 3). (E) Flow cytometry analysis of the cell cycle distribution and proliferation in PDAC cell lines treated with DMSO or Cal. All experiments were conducted in triplicate. ns: not significant, ∗ p < 0.05. Figure S2: THBS1 and CD47 expression and survival analysis in PDAC. (A) A protein‐interaction network illustrates the connections between THBS1, VDR, and other proteins. (B) Using GEPIA, transcriptomic analysis of PDAC tumor samples reveals the expression of THBS1 across a cohort, with a box plot indicating a correlation between high THBS1 levels and decreased disease‐free survival. (C) ELISA quantification of TGF‐β protein levels in the supernatant of aPSCs treated with medium (Ctr), DMSO, or 100 nM Cal, showing no significant differences. (D) Gene expression profile for CD47 across the same PDAC cohort, as analyzed using GEPIA, with a box plot illustrating the association between high CD47 levels and reduced disease‐free survival. (E) Western blot analysis of CD47 protein levels in PDAC tumor tissue (T) and adjacent normal tissue (N) from patients, with densitometry confirming higher CD47 expression in tumor tissue (n = 3). (F) qRT‐PCR analysis of LCAM expression in PDAC cells treated with aPSCs‐CM (Ctr), DMSO‐aPSCs‐CM, or Cal‐aPSCs‐CM, indicating no significant change in expression due to Cal treatment (n = 3). All experiments were conducted in triplicate. ns [file MI-2026-2632235-s001.docx]

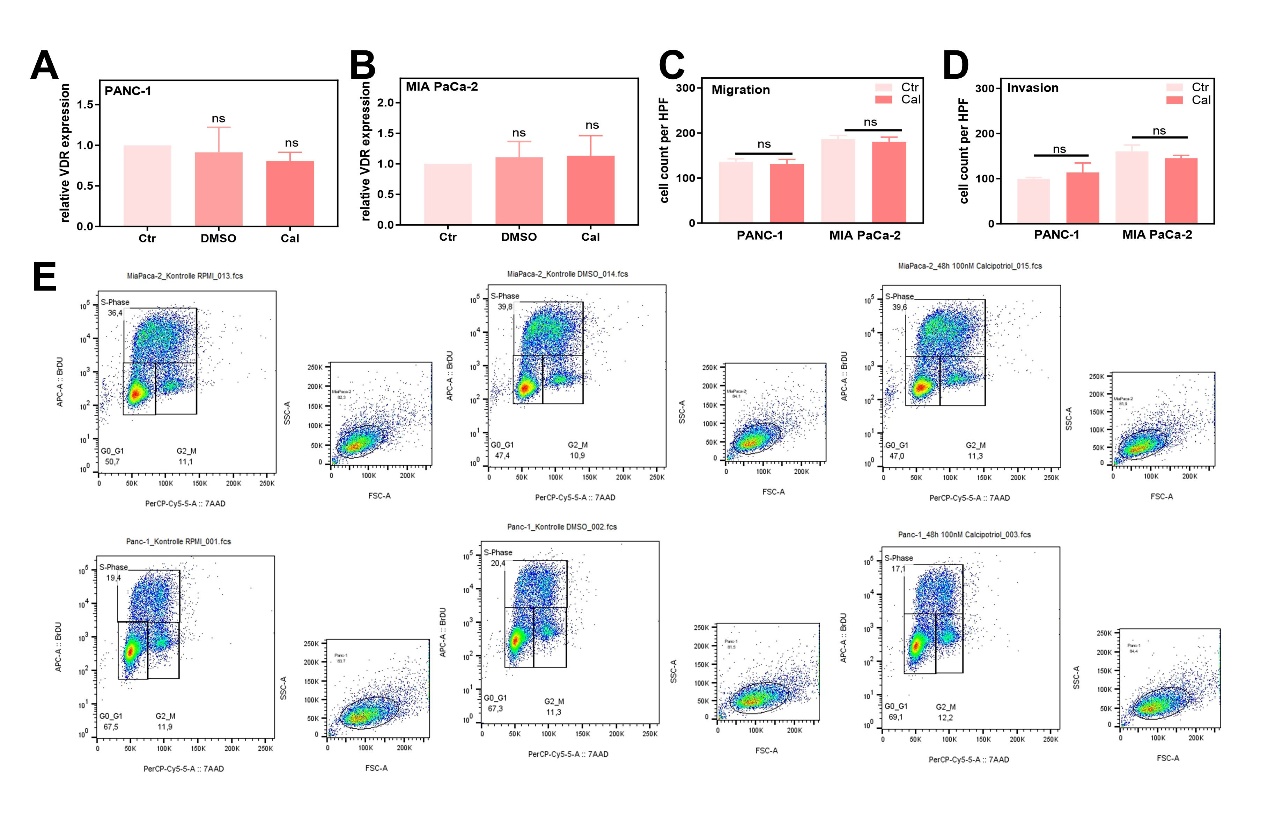


**Supplementary Figure 1. Evaluation of VDR Expression and Functional Assays in PDAC Cell Lines After Cal Treatment.** (A) Quantification of VDR expression in PANC-1 cells treated with control (RPMI1640), DMSO, or 100nM Cal. (B) Quantification of VDR expression in MIA PaCa-2 cells under similar treatment conditions (n=3). (C) Migration assay results for PANC-1 and MIA PaCa-2 cells, showing cell counts per high power field (HPF) with or without Cal treatment (n=3). (D) Invasion assay results for the same cell lines, indicating no significant difference in cell count per HPF between treatments (n=3). (E) Flow cytometry analysis of the cell cycle distribution and proliferation in PDAC cell lines treated with DMSO or Cal. All experiments were conducted in triplicate. ns: not significant, *p<0.05.


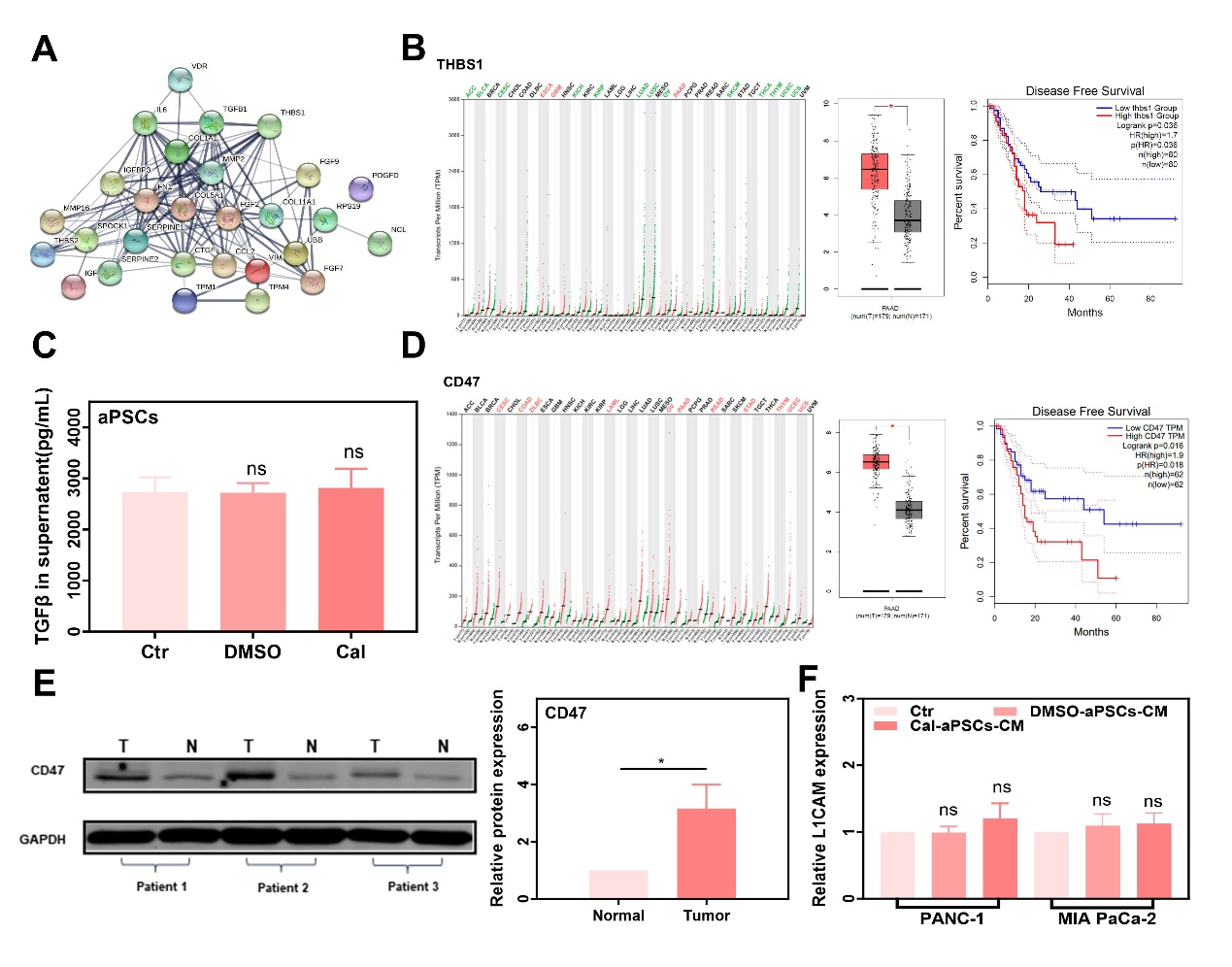


**Supplementary Figure 2. THBS1 and CD47 Expression and Survival Analysis in PDAC.** (A) A protein-interaction network illustrates the connections between THBS1, VDR, and other proteins. (B) Using GEPIA, transcriptomic analysis of PDAC tumor samples reveals the expression of THBS1 across a cohort, with a box plot indicating a correlation between high THBS1 levels and decreased disease-free survival. (C) ELISA quantification of TGF-β protein levels in the supernatant of aPSCs treated with medium (Ctr), DMSO, or 100nM Cal, showing no significant differences. (D) Gene expression profile for CD47 across the same PDAC cohort, as analyzed using GEPIA, with a box plot illustrating the association between high CD47 levels and reduced disease-free survival. (E) Western blot analysis of CD47 protein levels in PDAC tumor tissue (T) and adjacent normal tissue (N) from patients, with densitometry confirming higher CD47 expression in tumor tissue (n=3). (F) qRT-PCR analysis of LCAM expression in PDAC cells treated with aPSCs-CM (Ctr), DMSO-aPSCs-CM, or Cal-aPSCs-CM, indicating no significant change in expression due to Cal treatment (n=3). All experiments were conducted in triplicate. ns: not significant, *p<0.05.
